# Apolipoprotein E gene ε4ε4 is associated with elevated risk of primary open angle glaucoma in Asians: a meta-analysis

**DOI:** 10.1186/1471-2350-15-60

**Published:** 2014-05-19

**Authors:** Yong Wang, Yan-Feng Zhou, Bing-Ying Zhao, Zheng-Yu Gu, Shou-Ling Li

**Affiliations:** 1 Department of Ophthalmology, The First Affiliated Hospital of Anhui Medical University, 218 Jixi Road, Hefei 230022, Anhui, China

**Keywords:** Glaucoma, *APOE*, Genetic, Meta-analysis

## Abstract

**Background:**

Epidemiological studies have evaluated the association between Apolipoprotein E (*APOE*) gene ε2/ε3/ε4 polymorphism and glaucoma susceptibility. However, the published data are still inconclusive. The aim of the present study is to evaluate the impact of *APOE* gene ε2/ε3/ε4 polymorphism on glaucoma risk by using meta-analysis.

**Methods:**

A comprehensive literature search of PubMed, EMBASE, Cochrane, Elsevier Science Direct and CNKI databases was conducted to identify relevant articles, with the last report up to January 5, 2014. Pooled odds ratio (OR) and 95% confidence interval (CI) were used to assess the strength of association by using the fixed or random effect model.

**Results:**

Fifteen separate studies including 2,700 cases and 2,365 controls were included in the meta-analysis. We did not detect a significant association between *APOE* gene ε2/ε3/ε4 polymorphism and glaucoma in overall population (*P* > 0.0083). In Asians, we detected an association of the ε4ε4 genotype with elevated risk for glaucoma (OR = 5.22, 95% CI = 1.85-14.68, *P* = 0.002), mainly for primary open angle glaucoma (OR = 4.98, 95% CI = 1.75-14.20, *P* = 0.003).

**Conclusions:**

The meta-analysis suggests that *APOE* gene ε4ε4 may be associated with elevated risk for primary open angle glaucoma in Asians. However, more epidemiologic studies based on larger sample size, case–control design and stratified by ethnicity as well as types of glaucoma are suggested to further clarify the relationship between *APOE* gene ε2/ε3/ε4 polymorphism and genetic predisposition to glaucoma.

## Background

Glaucoma, a group of diseases causing optic neuropathy, is characterized by optic nerve head changes and visual field loss, and is the second leading cause of irreversible blindness across the world [1,2]. In 1995, the World Health Organization reported that 5.1 million people were bilaterally blind from glaucoma [1]. Glaucoma is expected to affect 79.6 million people worldwide by the year 2020 [2]. Its morbidity and prevalence make it a significant public health problem [1]. However, to date, the pathogenesis of glaucoma remains largely unknown, with both genetic and environmental factors contributing to the pathophysiology [1,3]. Over the last decade, the application of molecular genetic techniques to the study of glaucoma has been accelerated greatly, and the accumulating evidence supports an important role for genetics in determining risk for this disease [3].

Apolipoprotein E (*APOE*), which is a major apolipoprotein in the central nervous system, plays an important role in the uptake and redistribution of cholesterol within neuronal network [4]. *APOE* gene is located on human chromosome 19q13.2 and is polymorphic [5,6]. There are three common *APOE* isoforms E2, E3 and E4, encoded by different alleles (ε2, ε3 and ε4), which vary significantly in structure and functions [7]. *APOE* gene ε2/ε3/ε4 polymorphism has been associated with several neurodegenerative diseases such as Alzheimer's and Parkinson's diseases [8,9]. Therefore, it is possible that this polymorphism may be associated with glaucoma risk [10]. Recently, a number of studies have been conducted to investigate the association of *APOE* gene ε2/ε3/ε4 polymorphism with glaucoma risk [11-25]. However, the results are controversial with some reports showing positive association [13,15,18-21,24,25] while others showed no association [11,12,14,16,17,22,23]. The inconsistent conclusion may be due to the relatively small size of subjects, since small sample sized association studies could be very limited for efficient assessment of the association.

Integration of these studies may provide improved statistical power to detect the significance. Meta-analysis is a statistical procedure for combining the results of several studies to produce a single estimate of the major effect with enhanced precision [26]. Recently, two studies assessed the association of *APOE* gene ε2/ε3/ε4 polymorphism with primary open angle glaucoma (POAG) by using meta-analysis [27,28]. Their meta-analyses suggest that this polymorphism is not associated with POAG susceptibility. However, the results of their meta-analyses should be interpreted with caution due to some potential limitations. Therefore, in this study, we conducted a meta-analysis to derive a more precise estimation of the association between *APOE* gene ε2/ε3/ε4 polymorphism and the risk for glaucoma.

## Methods

### Search strategy and inclusion criteria

This meta-analysis was reported according to the Preferred Reporting Items for Systematic Reviews and Meta-Analysis (PRISMA) Statement, issued in 2009 (Additional file 1: Table S1). Electronic databases, including PubMed, Excerpta Medica Database (EMBASE), Cochrane, Elsevier Science Direct and China National Knowledge Infrastructure (CNKI) databases, were searched for identification of the studies on *APOE* gene polymorphisms and glaucoma published up to January 5, 2014. Search terms included ‘glaucoma’ and ‘apolipoprotein OR APOE OR Apo E OR ApoE’. There was no language restriction. Review articles and original papers were searched by hand for additional eligible studies. The following criteria were used to select the eligible studies: (a) evaluation of the association between *APOE* gene ε2/ε3/ε4 polymorphism and glaucoma risk; (b) an unrelated case–control study in which family members were excluded; (c) sufficient published data for estimating an odds ratio (OR) with 95% confidence interval (CI). When authors reported two or more publications on the same patient population, only the largest study was selected. Additionally, when a study reported the results on different subpopulations, we treated them as a separate study.

### Data extraction

Information was carefully extracted from all eligible publications independently by two investigators. Disagreement was resolved by discussion between the two investigators. The following data were collected from each study: the first author’s name, publication year, source of publication, ethnicity, sample size (numbers of cases and controls), types of glaucoma, and allele as well as genotype frequencies. Authors were contacted for further information when necessary.

### Statistical analysis

Pooled OR with 95% CI was used to assess the strength of association between *APOE* gene ε2/ε3/ε4 polymorphism and glaucoma risk. The significance of the pooled OR was determined by *Z* test. Genotype ε3ε3 is the most common genotype [29]. Thus, genotype ε3ε3 or allele ε3 is designated as reference category in the present study. We estimated the risk of the ε2 and ε4 alleles, compared with the ε3 allele. We also performed genotypic analyses (ε2 carriers versus ε3ε3, ε2ε2 versus ε3ε3, ε4 carriers versus ε3ε3 and ε4ε4 versus ε3ε3). ε2 carriers were defined as patients with the ε2ε2 and ε2/ε3 genotypes. ε4 carriers were defined as patients with the ε3ε4 and ε4ε4 genotypes. Genotype ε2ε4 was excluded from the genotypic analyses because of the opposite effect between ε2 and ε4 alleles [15,30]. In addition, we performed subgroup analyses by ethnicity (Cauasians and Asians) and types of glaucoma (POAG) when the data were available. Subgroup analyses were also performed by excluding those studies which did not fulfill Hardy-Weinberg equilibrium (HWE). We carried out sensitivity analysis by excluding one study at a time to explore whether the results were influenced by a specific study. Heterogeneity (between-study inconsistency) was investigated and measured using Cochran’s *Q* statistic and *I*^*2*^ statistic [31,32]. A *P* value greater than 0.10 indicated a lack of heterogeneity among studies, so the fixed effect model (Mantel-Haenszel method) was used to calculate pooled OR [33]. Otherwise, the random effect model (DerSimonian-Laird method) was used [34]. Publication bias was detected using Egger’s linear regression test by visual inspection of funnel plot [35]. A Chi square-test was used to evaluate the deviation from HWE in controls. All analyses were done with the software Review Manager (v4.2; Oxford, England) and Stata statistical software (v10.0; StataCorp, College Station, TX, USA), using two-sided *P* values. *P* values were Bonferroni adjusted to account for multiple testing. In overall analyses, *P* values below 0.0083 (0.05/6) were considered statistically significant, and, in subgroup analyses, *P* values below 0.0042 (0.05/12) were considered statistically significant.

## Results

### Characteristics of studies

**Figure 1:**
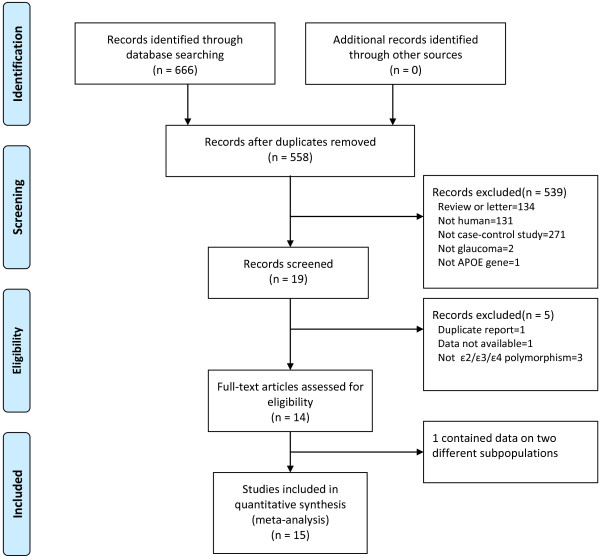
Flow diagram of the study selection process.

Characteristics of studies investigating the association of *APOE* gene ε2/ε3/ε4 polymorphism with glaucoma risk are presented in Table 1[11-24]. The study selection process is shown in Figure 1. There were 666 articles relevant to the searching terms (PubMed: 51; EMBASE: 95; Cochrane: 0; Elsevier Science Direct: 513; CNKI: 7). Nineteen studies examined the association between *APOE* gene polymorphisms and glaucoma. Three studies did not explore the ε2/ε3/ε4 polymorphism and were excluded [36-38]. One study was excluded due to duplicate report [39], and another study was excluded due to unavailable data [25]. Krumbiegel et al. [11] provided data on two different subpopulations (German and Italian), and, thus, each subpopulation was treated as a separate study. Finally, a total of fifteen separate studies including 2,700 cases and 2,365 controls were included in the present meta-analysis [11-24].

**Table 1:** **Characteristics of studies included in the meta**-**analysis of apolipoprotein E gene ε2**/**ε3**/ε4 polymorphism and glaucoma^*^

**Author/Year [Reference]**	**Ethnicity**	**Sample size (Case/control)**	**Glaucoma type**	**Number of cases**			**Number of controls**			**HWE *P*-value**
				**ε2**	**ε3**	**ε4**	**ε2**	**ε3**	**ε4**	
Krumbiegel 2010 [11]	Caucasian (German)	801(459/342)	PEXG	79	740	99	51	547	86	0.926
Krumbiegel 2010 [11]	Caucasian (Italian)	323(133/190)	PEXG	21	222	23	23	317	40	0.024
Saglar 2009 [12]	Caucasian	194(75/119)	POAG	13	126	11	10	204	24	0.832
Al-Dabbagh 2009 [13]	Asian	230(100/130)	POAG,PACG	0	181	19	0	249	11	0.969
Jia 2009 [14]	Asian	376(176/200)	POAG	34	278	40	35	329	36	0.897
Yuan 2007 [15]	Asian	162(105/57)	POAG,PACG	48	81	81	18	76	20	<0.0001
Zetterberg 2007 [16]	Caucasian	429(242/187)	POAG	50	376	58	42	289	43	0.923
Hu 2007 [17]	Asian	219(142/77)	POAG	17	229	38	11	129	14	0.409
Tamura 2006 [18]	Asian	105(28/77)	POAG	3	42	11	12	135	7	0.079
Lam 2006 [19]	Asian	700(400/300)	POAG	79	674	47	50	495	55	0.065
Mabuchi 2005 [20]	Asian	489(310/179)	POAG	16	567	37	18	302	38	0.104
Jünemann 2004 [21]	Caucasian	128(96/32)	POAG	19	146	27	9	40	15	0.012
Lake 2004 [22]	Caucasian	504(155/349)	POAG	28	229	53	56	534	108	0.316
Ressiniotis 2004 [23]	Caucasian	212(137/75)	POAG	35	199	40	16	114	20	NA^#^
Vickers 2002 [24]	Caucasian	193(142/51)	POAG	27	206	51	15	75	12	0.202

*HWE: Hardy-Weinberg equilibrium; PEXG: pseudoexfoliation glaucoma; POAG: primary open angle glaucoma; PACG: primary angle closure glaucoma; NA: not available. ^#^Only allele frequency was extracted from the study.

The allele and genotype frequencies of *APOE* gene ε2/ε3/ε4 polymorphism were extracted from fourteen separate studies [11-22,24]. Only allele frequency was extracted from the study by Ressiniotis et al. [23]. The results of HWE test for the distribution of the genotype in control population are shown in Table 1. The genotype distributions in eleven separate studies were in agreement with HWE. The genotype distributions in 3 separate studies were not in agreement with HWE. It was unavailable for the study by Ressiniotis et al. to perform HWE test. The fifteen studies consisted of eight Caucasian studies and seven Asian studies. Of these studies, two studies were conducted in patients with pseudoexfoliation glaucoma (PEXG), eleven studies were conducted in patients with primary open angle glaucoma (POAG), and two studies were conducted in patients with POAG and primary angle closure glaucoma (PACG).

### Quantitative synthesis

The results of the meta-analysis on the association between *APOE* gene ε2/ε3/ε4 polymorphism and risk of glaucoma are shown in Table 2. By pooling all the studies, *APOE* gene ε2/ε3/ε4 polymorphism was not associated with glaucoma risk (*P* > 0.0083). We detected significant between-study heterogeneity for the contrasts of ε2 versus ε3, ε2 carriers versus ε3ε3, ε4 versus ε3 and ε4 carriers versus ε3ε3 in overall population (*P* < 0.1). We carried out subgroup analyses by excluding those studies which did not fulfill HWE, and found similar results (*P* > 0.0042). Significant between-study heterogeneity for the contrasts of ε2 carriers versus ε3ε3, ε4 versus ε3 and ε4 carriers versus ε3ε3 was detected in HWE population (*P* < 0.1).

**Table 2:** **Meta**-**analysis of the association between apolipoprotein E gene ε2**/**ε3**/ε4 polymorphism and glaucoma^*^

**Groups**		**Sample size**		**Number of Studies**	**Test of association**				**Test of heterogeneity**		
		**Case**	**Control**		***OR *** **(*95 *% *CI*)**	***Z***	***P*-*value***	***Model***	***χ***^***2***^	***P*-*value***	***I***^***2***^**(%)**
Overall	ε2 vs ε3	4765	4201	15	1.08(0.88-1.32)	0.74	0.46	R	20.83	0.08	37.6
	ε2 carriers vs ε3ε3	2001	1804	14	1.02(0.79-1.33)	0.18	0.86	R	21.18	0.05	43.3
	ε2ε2 vs ε3ε3	1688	1541	14	1.11(0.54-2.28)	0.28	0.78	F	3.51	0.83	0.0
	ε4 vs ε3	4931	4364	15	1.14(0.86-1.51)	0.92	0.36	R	58.45	<0.0001	76.0
	ε4 carriers vs ε3ε3	2146	1955	14	1.15(0.82-1.59)	0.81	0.42	R	50.80	<0.0001	74.4
	ε4ε4 vs ε3ε3	1704	1549	14	1.66(0.99-2.78)	1.92	0.05	F	14.05	0.23	21.7
Controls in HWE	ε2 vs ε3	3994	3588	11	1.03(0.87-1.22)	0.36	0.72	F	11.02	0.27	18.3
	ε2 carriers vs ε3ε3	1790	1597	11	0.93(0.71-1.22)	0.54	0.59	R	15.50	0.08	41.9
	ε2ε2 vs ε3ε3	1525	1359	11	1.34(0.62-2.90)	0.73	0.46	F	2.05	0.92	0.0
	ε4 vs ε3	4112	3722	11	1.10(0.83-1.47)	0.69	0.49	R	33.90	0.0002	70.5
	ε4 carriers vs ε3ε3	1900	1723	11	1.04(0.77-1.42)	0.26	0.79	R	29.86	0.0009	66.5
	ε4ε4 vs ε3ε3	1533	1367	11	1.41(0.80-2.50)	1.18	0.24	F	10.35	0.32	13.1
Caucasian	ε2 vs ε3	2516	2342	8	1.08(0.89-1.31)	0.74	0.46	F	7.70	0.36	9.1
	ε2 carriers vs ε3ε3	996	963	7	1.07(0.84-1.36)	0.55	0.58	F	6.65	0.35	9.8
	ε2ε2 vs ε3ε3	821	813	7	0.92(0.41-2.09)	0.20	0.84	F	2.74	0.60	0.0
	ε4 vs ε3	2606	2468	8	0.96(0.81-1.13)	0.51	0.61	F	7.89	0.34	11.3
	ε4 carriers vs ε3ε3	1079	1076	7	0.93(0.75-1.14)	0.73	0.46	F	4.62	0.59	0.0
	ε4ε4 vs ε3ε3	823	822	7	0.92(0.47-1.77)	0.26	0.79	F	5.52	0.36	9.3
Asian	ε2 vs ε3	2249	1859	7	1.07(0.71-1.62)	0.32	0.75	R	13.05	0.02	61.7
	ε2 carriers vs ε3ε3	1005	841	7	0.95(0.56-1.63)	0.18	0.86	R	14.41	0.01	65.3
	ε2ε2 vs ε3ε3	867	728	7	2.08(0.45-9.63)	0.94	0.35	F	0.04	0.98	0.0
	ε4 vs ε3	2325	1896	7	1.53(0.83-2.84)	1.36	0.17	R	48.44	<0.0001	87.6
	ε4 carriers vs ε3ε3	1067	879	7	1.56(0.77-3.13)	1.24	0.21	R	45.75	<0.0001	86.9
	ε4ε4 vs ε3ε3	881	727	7	5.22(1.85-14.68)	3.13	0.002	F	2.95	0.71	0.0
POAG (Caucasian)	ε2 vs ε3	1454	1404	6	1.02(0.80-1.30)	0.13	0.90	F	6.95	0.22	28.1
	ε2 carriers vs ε3ε3	522	549	5	0.99(0.72-1.37)	0.05	0.96	F	5.92	0.20	32.5
	ε2ε2 vs ε3ε3	426	457	5	0.79(0.26-2.44)	0.41	0.68	F	0.56	0.76	0.0
	ε4 vs ε3	1522	1478	5	1.04(0.84-1.28)	0.34	0.74	F	6.56	0.26	23.8
	ε4 carriers vs ε3ε3	581	613	5	1.01(0.76-1.33)	0.05	0.96	F	3.59	0.47	0.0
	ε4ε4 vs ε3ε3	430	463	5	1.24(0.55-2.77)	0.52	0.60	F	3.05	0.38	1.6
POAG (Asian)	ε2 vs ε3	2091	1859	7	0.96(0.75-1.22)	0.34	0.73	F	6.27	0.28	20.2
	ε2 carriers vs ε3ε3	953	841	7	0.78(0.48-1.27)	1.00	0.32	R	10.68	0.06	53.2
	ε2ε2 vs ε3ε3	827	728	7	2.08(0.45-9.63)	0.94	0.35	F	0.04	0.98	0.0
	ε4 vs ε3	2149	1896	7	1.45(0.82-2.55)	1.29	0.20	R	37.92	<0.0001	84.2
	ε4 carriers vs ε3ε3	1000	879	7	1.45(0.76-2.76)	1.13	0.26	R	36.86	<0.0001	83.7
	ε4ε4 vs ε3ε3	838	727	7	4.98(1.75-14.20)	3.01	0.003	F	3.01	0.70	0.0

^*^POAG: primary open angle glaucoma; HWE: Hardy-Weinberg equilibrium; vs: versus; OR: odds ratio; CI: confidence interval; F: fixed effect model; R: random effect model.

When stratified by ethnicity, no association was found between *APOE* gene ε2/ε3/ε4 polymorphism and risk of glaucoma in Caucasians (*P* > 0.0042). No significant between-study heterogeneity was found in Caucasians (*P* > 0.1). In Asians, we detected an association of the ε4ε4 genotype with elevated risk for glaucoma (OR = 5.22, 95%CI = 1.85-14.68, *P* = 0.002). But we did not find significant association of *APOE* gene ε2/ε3/ε4 polymorphism with glaucoma in other models (*P* > 0.0042). Significant between-study heterogeneity was found for the contrasts of ε2 versus ε3, ε2 carriers versus ε3ε3, ε4 versus ε3 and ε4 carriers versus ε3ε3 in Asians (*P* < 0.1).

In subgroup analysis by types of glaucoma, we also detected an association of the ε4ε4 genotype with elevated risk for POAG in Asians (OR = 4.98, 95% CI = 1.75-14.20, *P* = 0.003), but not for POAG in Caucasians (*P* > 0.0042). In POAG population (Asians), we detected significant between-study heterogeneity for the contrasts of ε2 carriers versus ε3ε3, ε4 versus ε3 and ε4 carriers versus ε3ε3 (*P* < 0.1).

### Potential publication bias and sensitivity analysis

For most of comparisons, the shapes of the funnel plots did not reveal any evidence of obvious asymmetry (funnel plots not shown), and these results were further supported by analysis via Egger’s linear regression test (Table 3). We only found publication bias for the contrasts of ε4 carriers versus ε3ε3 in overall population and HWE population, ε4 versus ε3 as well as ε4 carriers versus ε3ε3 in Asians and POAG (Asians) subgroup (*P* < 0.05). Additionally, Egger’s test was not applied in the contrast of ε2ε2 versus ε3ε3 in Asians and the contrasts of ε2ε2 versus ε3ε3 as well as ε4 carriers versus ε3ε3 in POAG (Asians) subgroup due to the rare frequency of homozygous mutation.

**Table 3:** **Egger**’s linear regression test to measure the funnel plot asymmetry^*^

**Groups**	**Y axle intercept: *a *** **(*95 *% *CI*)**					
	**Overall**	**Controls in HWE**	**Caucasian**	**Asian**	**POAG (Caucasian)**	**POAG (Asian)**
ε2 vs ε3	−0.66(−3.09-1.77)	−0.93(−3.39-1.54)	−0.22(−3.69-3.23)	−1.06(−6.79-4.66)	0.03(−5.81-5.87)	−1.68(−4.48-1.13)
ε2 carriers vs ε3ε3	−0.03(−2.36-2.29)	−1.38(−4.14-1.38)	0.55(−2.73-3.83)	−0.50(−5.90-4.90)	1.14(−4.80-7.08)	−2.07(−7.60-3.46)
ε2ε2 vs ε3ε3	−0.94(−5.04-3.15)	−0.94(−5.04-3.15)	−3.15(−8.03-1.73)	NA	−2.28(−16.07-11.52)	NA
ε4 vs ε3	2.81(−0.76-6.39)	3.21(−0.14-6.56)	−0.45(−3.63-2.73)	7.63(0.14-15.12)	−1.60(−6.29-3.08)	7.26(2.85-11.66)
ε4 carriers vs ε3ε3	3.49(0.56-6.41)	3.23(0.16-6.31)	0.38(−2.34-3.11)	7.73(3.29-12.18)	0.35(−4.70-5.40)	6.54(3.07-10.01)
ε4ε4 vs ε3ε3	−2.15(−5.52-1.22)	−2.18(−6.29-1.93)	−2.35(−6.03-1.32)	2.01(−1.14-5.15)	−2.26(−6.69-2.17)	NA

^*^POAG: primary open angle glaucoma; HWE: Hardy-Weinberg equilibrium; vs: versus; CI: confidence interval; NA: not available.

We carried out sensitivity analysis by excluding one study at a time to explore whether the positve result of POAG (Asians) subgroup were influenced by a specific study. Sensitivity analysis indicated that a significant variation in combined ORs by excluding the study by Al-Dabbagh et al. (OR = 4.01, 95% CI = 1.29-12.46, *P* = 0.02) or the study by Yuan et al. (OR = 4.04, 95% CI = 1.30-12.50, *P* = 0.02) or the study by Tamura et al. (OR = 4.62, 95% CI = 1.54-13.90, *P* = 0.006).

## Discussion

Recently, two studies assessed the association of *APOE* gene ε2/ε3/ε4 polymorphism with POAG by using meta-analysis [27,28]. Their meta-analyses suggest that this polymorphism is not associated with POAG susceptibility. However, the results of their meta-analyses should be interpreted with caution due to some limitations. In the study by Song et al. [27], the authors did not include all available published studies. Five eligible studies were not include in the meta-analysis [13,15,17,21,23]. Meanwhile, two studies contained overlapping data [19,39], and only the largest study [19] should be selected for the analysis. In another study by Wang et al. [28], there were many mistakes in the data extraction (see Supplementary Table two in their article). For example, the authors confused the cases and controls for the study by Zetterberg et al. [16]. The data provided by the authors for the study by Vickers et al. [24] were not in line with the data provided by Vickers et al. in their original publication. Close inspection of the data in the study by Jia et al. [14] revealed that the number of the ε2, ε3 and ε4 alleles in cases should be 34, 278 and 40, but not 34, 280 and 38 in the study by Wang et al.. These limitations distorted the results of their meta-analysis. In the present study, we systemically reviewed all available published studies and performed a meta-analysis to derive a more precise estimation of the association between *APOE* gene ε2/ε3/ε4 polymorphism and susceptibility to glaucoma. Our meta-analysis included fifteen separate studies involving 2,700 cases and 2,365 controls. In Asians, we detected an association of the ε4ε4 genotype with elevated risk for glaucoma, mainly for POAG. Thus, the ε4ε4 genotype may be associated with elevated risk for POAG in Asians.

Glaucoma is a term describing a group of ocular disorders with multi-factorial etiology united by a clinically characteristic intraocular pressure-associated optic neuropathy [1,40]. This disease often goes undetected. In the United States, glaucoma is responsible for 9-12% of blindness, and impacts up to three million Americans, but only half of those are aware they have it [41]. Glaucoma is a neurodegenerative disease, which is often associated with high intraocular pressure [10,42]. In this disease, there are cell bodies (retinal ganglion cells) and nerve fibers (optic nerve axons) that are vulnerable to degeneration and amenable to protection. Neurodegeneration occurs in the retinal ganglion neuronal cell bodies, their axons in the optic nerve, the lateral geniculate nucleus, optic radiation, and visual cortex [43,44]. *APOE* plays a key role in lipid metabolism, cholesterol transport, as well as protein synthesis, and is also involved in numerous other functions, including tissue repair, cell growth and differentiation, and immune response and regulation [4,45]. *APOE* gene ε2/ε3/ε4 polymorphism has been associated with a number of neurodegenerative diseases such as Alzheimer's and Parkinson's diseases [8,9], raising the possibility that this polymorphism might predispose to neurodegeneration of the retinal ganglion cells and the optic nerve axons in glaucoma. In the study, we found that the ε4ε4 genotype is associated with elevated risk for POAG in Asians. The extracellular accumulation of insoluble fibrillar peptides in brain parenchyma and vessel walls as amyloid is the hallmark of neurodegenerative diseases [46]. Amyloid β-peptide (Aβ) deposition in senile plaques and cerebral vessels is a neuropathological feature of Alzheimer’s disease [47]. There is evidence that the amount of histologically identified Aβ in vessels and plaques in the cerebral cortex of patients with Alzheimer’s disease is a direct function of their APOE genotype, and subjects with ε4ε4 genotype have an increased amyloid deposits in vessels and density of strongly Aβ-immunoreactive plaques than those with ε3ε3 genotype [47]. Numerous similarities exist between glaucoma and Alzheimer's diseases [10], and, thus, the evidence supports our results. However, the exact function of *APOE* gene ε2/ε3/ε4 polymorphism remains largely unknown, and studies of the functional implications of this polymorphism are still needed in the future. Additionally, we found that the ε4ε4 genotype is associated with elevated risk for POAG in Asians, but not in Caucasians, which indicated that the genetic background of Asians and Caucasians is different.

Some limitations of this meta-analysis should be acknowledged. A first consideration is that we found publication bias for some contrasts. A second consideration is that significant between-study heterogeneity was found in some comparisons, which may be distorting the results of meta-analysis. In the subgroup analysis by ethnicity, the heterogeneity disappeared among Caucasians. The heterogeneity may have resulted from differences in the subjects’ genetic backgrounds. Additionally, different types of glaucoma may contribute to the heterogeneity. But most of studies did not provide glaucoma subtype information (e.g., normal tension glaucoma or high tension glaucoma). Third, though we detected an association of the ε4ε4 genotype with elevated risk for glaucoma (with POAG and PCAG combined) in Asians, only two studies were conducted in patients with PACG (109 patients). Thus, further studies exploring the association between *APOE* gene ε2/ε3/ε4 polymorphism and PACG risk are still needed. Fourth, sensitivity analysis indicated that a significant variation in combined ORs by excluding some studies. Finally, our results are based on unadjusted estimates, which may be distorting the results of meta-analysis. A more precise analysis stratified by environmental factors could be performed if individual data were available.

## Conclusions

In summary, the present meta-analysis suggests that *APOE* gene ε4ε4 may be associated with elevated risk for POAG in Asians. However, more epidemiologic studies based on larger sample size, case–control design and stratified by ethnicity as well as types of glaucoma are suggested to further clarify the relationship between *APOE* gene ε2/ε3/ε4 polymorphism and genetic predisposition to glaucoma.

## Abbreviations

APOE: Apolipoprotein E; Aβ: Amyloid β-peptide; CI: Confidence interval; CNKI: China National Knowledge Infrastructure; EMBASE: Excerpta Medica Database; HWE: Hardy-Weinberg equilibrium; OR: Odds ratio; PACG: Primary angle closure glaucoma; PEXG: Pseudoexfoliation glaucoma; POAG: Primary open angle glaucoma; PRISMA: Preferred reporting items for systematic reviews and meta-analysis.

## Competing interests

The authors declare that they have no competing interest.

## Authors’ contributions

S-LL, YW, Y-FZ, B-YZ and Z-YG conceived and designed the study. S-LL, YW, Y-FZ, B-YZ and Z-YG took part in literature review and data extraction. S-LL and YW were involved in software used and data analysis. S-LL, YW, Y-FZ, B-YZ and Z-YG wrote the paper. All authors read and approved the final manuscript.

## Funding

This work was supported by Anhui Provincial Natural Science Foundation (1408085MH158).

## Pre-publication history

The pre-publication history for this paper can be accessed here:

http://www.biomedcentral.com/1471-2350/15/60/prepub

## Supplementary Material

Additional file 1: Table S1 PRISMA Checklist. Click here for file
